# Case report: A giant bilateral inguinal hernia requiring artificial mesh and multi-stage surgery in infancy; hernioplasty with silo placement to prevent acute compartment syndrome

**DOI:** 10.3389/fped.2022.1030934

**Published:** 2022-11-10

**Authors:** Yoichi Nakagawa, Satoshi Makita, Hiroo Uchida, Akinari Hinoki, Chiyoe Shirota, Wataru Sumida, Hizuru Amano, Masamune Okamoto, Aitaro Takimoto, Akihiro Yasui, Seiya Ogata, Shunya Takada, Daiki Kato, Yousuke Gohda, Guo Yaohui

**Affiliations:** ^1^ Department of Pediatric Surgery, Nagoya University Graduate School of Medicine, Nagoya, Japan; ^2^ Department of Rare/Intractable Cancer Analysis Research, Nagoya University Graduate School of Medicine, Nagoya, Japan

**Keywords:** inguinal hernia, silo placement, abdominal compartment syndrome, infants, muti-stage surgery

## Abstract

Hernioplasty for giant inguinal hernias can cause abdominal compartment syndrome (ACS) in adults but rarely does in infants. We encountered a case of a giant bilateral inguinal hernia in infancy complicated by ACS after hernioplasty. Silo placement *via* a skin incision effectively treated ACS, after which the abdominal wall was safely closed. Hernioplasty performed early in the clinical course can help expand the abdominal cavity and avoid ACS. Thus, hernioplasty should be performed earlier if the hernia size in the flank space gradually increases.

## Introduction

In adults, giant inguinal hernias are generally treated by hernioplasty, which can cause abdominal compartment syndrome (ACS). However, this type of hernia is rare in children, and to our knowledge, there are no reports of infants with ACS after hernioplasty. We present a case of a giant bilateral inguinal hernia in an infant complicated by ACS after hernioplasty but treated with multi-stage surgery using a silo.

## Case description

**Figure 1:**
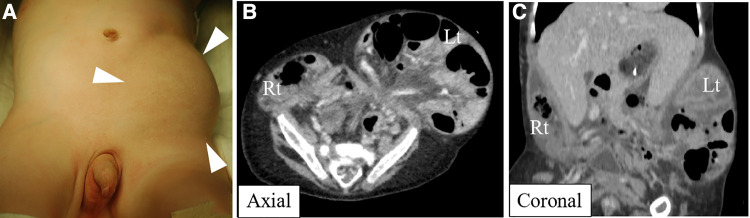
Physical examination revealed left flank swelling (**A**), and computed tomography revealed a bilateral inguinal hernia extending into both flanks between the abdominal wall and the skin (**B,C**).

A seven-month-old male patient was referred to our institution for a hernioplasty. The patient was born at a gestational age of 37 weeks, weighing 2,146 g, and was diagnosed with arthrogryposis multiplex congenita, bilateral cryptorchidism, and left flank swelling after birth. Left flank swelling was diagnosed as a left inguinal hernia. The patient often developed bradycardia, and sick sinus syndrome (SSS) was diagnosed at the age of 1 month. Progression of the left inguinal hernia was observed, and its size gradually increased (Figure 1A). Furthermore, computed tomography and abdominal radiography showed that the left inguinal hernia extended into the left flank between the body wall and skin (Figures 1B,C). The patient required pacemaker placement to treat the SSS, but the left inguinal hernia limited the access route. Therefore, hernioplasty and bilateral cryptorchidism were performed for pacemaker placement at 7 months.

## Diagnostic assessment

**Figure 2:**
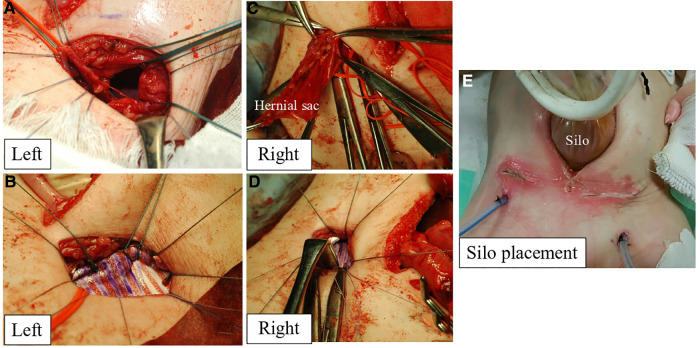
The left herniated orifice (**A**) was closed with GORE^®^ DUALMESH^®^ biomaterial and non-absorbable threads by suturing with the pubic tubercle, transversus abdominis fascia, and iliopubic tract (**B**). The right herniated orifice (**C**) was closed with GORE^®^ DUALMESH^®^ Biomaterial (**D**). After reducing prolapsed intestine into the abdominal cavity, the silo was placed at the midline incision (**E**).

First, a lower-midline incision was made. Then, we attempted to return a large amount of the small intestine that had prolapsed from the internal inguinal ring into the abdominal cavity through the midline wound. However, there was strong adhesion to the hernia sac; thus, an additional 3 cm of skin above the left internal inguinal ring was incised to identify the hernia sac. Next, the external oblique fascia was incised from the left inguinal incision, and the spermatic cord structure and hernial sac were identified. The small intestine was detached from the hernial sac, and the intestinal tract was returned to the abdominal cavity. The left testicular blood vessels and ductus deferens were identified and then tracked to find the left testis. The left ductus deferens was strongly adhered to the hernia sac with kinking; therefore, it was dissected *via* ligation. A left orchiopexy was performed after adhesiolysis. After removing the hernial sac, a large, herniated orifice was identified (Figure 2A). The transversus abdominis muscle was also fragile and extended. Thus, 5 cm × 2.5 cm GORE® DUALMESH® Biomaterial (W.L. Gore & Associates G.K., Tokyo, Japan) was used to close the herniated orifice with non-absorbable threads by suturing with the pubic tubercle, transversus abdominis fascia, and iliopubic tract (Figure 2B). The same manipulation was performed in the right inguinal region as in the left. The right testis was present at the caudal side of the herniated orifice. A right orchiopexy was performed after adhesiolysis. However, malrotation was diagnosed after the entire small intestine was returned to the abdominal cavity. Thus, the Ladd bands were dissected.

The right herniated orifice was also large, requiring 4 cm × 2.5 cm GORE® DUALMESH® Biomaterial to close the orifice (Figures 2C,D). All prolapsed intestines were reduced into the abdominal cavity. However, the respiratory tidal volume decreased owing to a large amount of previously prolapsed intestine and intestinal tract edema, indicating ACS. Hence, the silo was placed at the midline incision (Figure 2E). The drains were inserted into the subcutaneous space where the prolapsed intestinal tract had developed.

The operative time was 375 min, with a blood loss of 79 ml. After surgery, the prolapsed intestine was gradually reduced into the abdominal cavity through the silo. Wound closure was performed on postoperative day 10 after confirming that complete reduction did not cause respiratory and circulatory failure. Surgical site infection occurred after surgery. Still, the patient was discharged on postoperative day 19 without further complications.

## Discussion

A giant inguinal hernia is an inguinal hernia extending below the midpoint of the inner thigh when the patient is in a standing position (1). Our patient could not stand; however, the size of the hernia was equivalent to that of a defined giant inguinal hernia. Notably, in our case, the hernia extended into the flank space between the body wall and skin, presumably because of the patient's arthrogryposis multiplex congenita comorbidity, which prevented the herniated organs from extending into the inguinal canal to the scrotum due to the hip contracture. However, the prolapsed organs extend into the flank space, a more fragile space than the contracted groin area. Thus, we concluded that the small abdominal cavity volume, contracted groin and abdominal wall, and relatively fragile subcutaneous tissue at the flank were the causes. We also assumed that the hernia was related to arthrogryposis multiplex congenita, which is associated with abdominal wall defects and inguinal hernia (2). Therefore, the patient possibly had a congenital abdominal wall defect combined with an inguinal hernia.

ACS can occur when hernioplasty is performed in giant prolapsed organs. ACS presents with organ ischemia and respiratory and circulatory failure due to an elevated diaphragm and abdominal vessel compression. In adults, ACS is defined as organ dysfunction with an intra-abdominal pressure (IAP) of >20 mmHg (3). However, in pediatric patients, a sustained IAP >10 mmHg with organ dysfunction indicates ACS (4). When ACS occurs, surgical decompression is the gold standard (5). We preoperatively evaluated whether ACS occurred under general anesthesia, finding that the tidal volume sometimes decreased slightly. Hence, we predicted that primary hernioplasty might be challenging to perform. During the operation, we checked for ACS by closing the wound, finding that wound closure was impossible because of respiratory failure. Thus, the giant bilateral inguinal hernia, intestinal edema induced by the long operation time, the small abdominal cavity, and abdominal wall rigidity due to arthrogryposis multiplex congenita were risk factors for ACS in this case.

Our treatment strategy for this case was reasonable. However, we debated when to perform the hernioplasty. The hernia was diagnosed at birth and gradually progressed, eventually requiring multi-stage hernioplasty. We spent approximately six hours completing the hernioplasty due to severe adhesions. However, this may have been easier if the hernia had been smaller. Nonetheless, in this case, the hernia gradually increased in size, and the prolapsed organs extended into the flank above the umbilicus level at 3 months. Considering the patient's hip contraction and SSS, we monitored the progression until gastrointestinal symptoms occurred. However, the hernia was large enough to prevent pacemaker placement and required hernioplasty.

Hernioplasty performed early in the clinical course can help expand the abdominal cavity; in such cases, ACS does not occur. Thus, hernioplasty should be performed earlier if the hernia size in the flank space gradually increases. Presumably, when the intestine prolapses into the scrotum through the inguinal canal, the abdominal pressure distributes stress equally across the abdominal cavity, inguinal canal, and scrotum; usually, this occurs in a normal inguinal hernia. However, abdominal pressure does not effectively put pressure on the abdominal wall when the intestine prolapses into the flank space. Instead, it puts pressure on the subcutaneous tissue above the abdominal wall fascia, creating space in this area. This space prevents increased abdominal pressure, leading to a small abdominal cavity. To our knowledge, none have reported this type of inguinal hernia. However, the mechanism is similar to that of omphalocele, where the abdominal cavity size does not increase because abdominal pressure escapes to the omphalocele. Thus, the pressure is not applied to the abdominal wall.

## Conclusion

We encountered a case of a giant bilateral inguinal hernia requiring multi-stage hernioplasty with silo placement and wound closure to prevent ACS. Silo placement *via* a skin incision effectively prevented ACS.

## Data Availability

The original contributions presented in the study are included in the article/Supplementary Material, further inquiries can be directed to the corresponding author/s.
